# Vocal production learning in mammals revisited

**DOI:** 10.1098/rstb.2020.0244

**Published:** 2021-10-25

**Authors:** Vincent M. Janik, Mirjam Knörnschild

**Affiliations:** ^1^ Scottish Oceans Institute, School of Biology, University of St Andrews, St Andrews KY16 8LB, UK; ^2^ Museum für Naturkunde, Leibniz-Institute for Evolution and Biodiversity Science, Berlin, Germany; ^3^ Animal Behavior Lab, Freie Universität, Berlin, Germany; ^4^ Smithsonian Tropical Research Institute, Balboa, Ancón, Panama

**Keywords:** vocal communication, Cetacea, Pinnipedia, Chiroptera, elephants, primates

## Abstract

Vocal production learning, the ability to modify the structure of vocalizations as a result of hearing those of others, has been studied extensively in birds but less attention has been given to its occurrence in mammals. We summarize the available evidence for vocal learning in mammals from the last 25 years, updating earlier reviews on the subject. The clearest evidence comes from cetaceans, pinnipeds, elephants and bats where species have been found to copy artificial or human language sounds, or match acoustic models of different sound types. Vocal convergence, in which parameter adjustments within one sound type result in similarities between individuals, occurs in a wider range of mammalian orders with additional evidence from primates, mole-rats, goats and mice. Currently, the underlying mechanisms for convergence are unclear with vocal production learning but also usage learning or matching physiological states being possible explanations. For experimental studies, we highlight the importance of quantitative comparisons of seemingly learned sounds with vocal repertoires before learning started or with species repertoires to confirm novelty. Further studies on the mammalian orders presented here as well as others are needed to explore learning skills and limitations in greater detail.

This article is part of the theme issue ‘Vocal learning in animals and humans’.

## 1. Introduction

Vocal production learning, the ability to modify the structure of vocalizations as a result of hearing those of conspecifics or sometimes other species, either live or from a recording [1], has received concentrated research attention in birds since the advent of the sound spectrograph in the 1950s [2]. In mammals, evidence for this ability was less forthcoming. In 1997, Janik & Slater [3] summarized evidence for vocal learning in mammals for the first time, with an updated version focusing on vocal traditions published 6 years later [4]. Since then, review chapters have been specific to different mammalian orders, with the most comprehensive compilation published in 2014 [5–8]. One of the key issues in all of these reviews was what kind of evidence provides sufficient and satisfactory proof of vocal production learning. The most challenging problem is often to exclude usage learning, the ability to produce an already existing call or song type in a new context [1]. Seemingly novel vocalizations are best compared against baseline recordings from before their presentation to an individual to evaluate novelty. This clearly is easier when animals copy other species such as human speech sounds or even non-biological or artificially generated noise as sometimes used in experimental studies. Often multiple studies investigating the same species provide the best evidence for vocal production learning.

**Figure 1.**
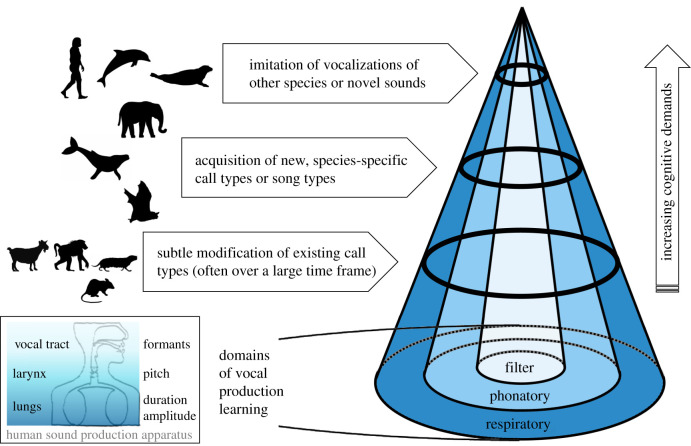
Different forms of vocal production learning. Vocal production learning is not a dichotomous trait but arranged on a continuum [13]. Manifestations of vocal production learning range from subtle modifications of existing call or song types to the imitation of vocalizations of other species or novel sounds. Sketches provide graphic references to mammalian vocal production learners covered in our review, and their position on the continuum represents our evaluation of their vocal production learning abilities. Three domains of vocal production learning (respiratory, phonatory and filter learning), their association with the sound producing apparatus, and the resulting signal characteristics are depicted as well. Most of the examples covered in our review concern phonatory learning. Note that different mammals can have vastly different sound production mechanisms (the human apparatus serves as a familiar example). Figure modified with permission from Scharff *et al.* [14]. (Online version in colour.)

Another challenge in the study of vocal learning is the tremendous variety of sound production mechanisms and techniques among animals. Birds and mammals employ similar mechanisms to produce sounds, but birds use a syrinx capable of producing two sounds at the same time while mammals usually use a larynx that is structurally different and does not have this dual voice capability [9]. Within mammals, the larynx is widely used, but in some cases, different structures take over. Odontocetes produce sounds with specifically evolved phonic lips in their nasal passages [10] and in elephants, the trunk may be used as a sound source [11]. In primates, lip smacking or unvoiced speech sounds are created by using parts of the mouth [12]. Janik & Slater [1] distinguished between effects of the respiratory, phonatory and filter system on vocalizations (figure 1). They highlighted that control over the respiratory system can influence the source level and duration of a vocalization, while only the phonatory and filter systems can have a substantial influence on spectral structure. However, changes in the respiratory system can also alter frequency parameters as in amplitude modulations adding side bands to signals or increased source levels leading to subtle increases in fundamental frequency [15–17]. Furthermore, the influence of the filter system which affects parameters of sounds after they have been produced by the phonatory system can be substantial, such as in human vowel production. Most examples given in our review appear to be cases of phonatory control, i.e. control over changes in frequency parameters that are indicative of direct control over the larynx or other production mechanisms used unless stated otherwise.

In our review, we revisit vocal production learning in mammals to summarize evidence published in the last 25 years. Only for species that have had no superseding evidence published since 1997 will we cite the earlier literature to provide a complete overview of this ability in mammals. Thus, this and the 1997 review should be read in conjunction if interested in complete coverage of the subject. Apart from studies that clearly show the copying of novel sounds (such as in copying of other species) or copying of different call or song types in different experimental groups of animals, we also summarize data on vocal convergence (figure 1). Convergence is often relatively subtle, usually affecting individual parameters within a call or song type. The underlying mechanisms of convergence are often unclear since different animals could use the same version of a vocalization because they are in the same motivational or physiological state (e.g. fearful animals often produce vocalizations with higher fundamental frequencies). In such cases, learning does not need to be involved. If learning is involved, it could be usage rather than production learning since the converged versions of vocalizations are rarely novel. Nevertheless, we think that convergence deserves further attention in the context of vocal learning recognizing that the delineation between different sound types is not always clear. Vocal learning skills can be restricted to specific parameter modifications or allow for copying of different species with a range of skills in between [13]. On this continuum, convergence could indicate a limited production learning ability. We therefore include studies in which at least two different, independent experimental groups show convergence towards different acoustic models as potential evidence for vocal production learning [13]. Ideally, these models are provided by the experimenter but studies in which groups converge on different group calls using sounds of group members as models can give similar evidence. It is important to note, however, that such evidence is only convincing when coming from highly controlled studies. Parameters like food availability, pressure from predator or competitor species, and group composition can create differences in stress or motivation of group members, potentially leading to acoustic differences without the influence of vocal production learning.

To be complete, we also report deafening and isolation studies, but we consider their interpretation to be problematic. To infer vocal production learning, it is not sufficient to show that the vocalizations of deafened or isolated animals develop abnormally. The abnormal development could also be caused by stress and/or sensory deprivation; the latter is especially relevant for echolocating taxa. Unlike Janik & Slater [3], we will not include data on dialects or geographic variation unless vocal learning has been demonstrated since such variation can arise from vocal learning as well as a multitude of other factors, including founder effects, habitat differences influencing vocalization choice or through genetic drift. Similarly, we do not include studies describing developmental changes unless vocal production learning rather than maturation or usage learning has been demonstrated or claimed. What we aim for here is to summarize the best available evidence for vocal learning in mammals.

## 2. Cetaceans

**Figure 2.**
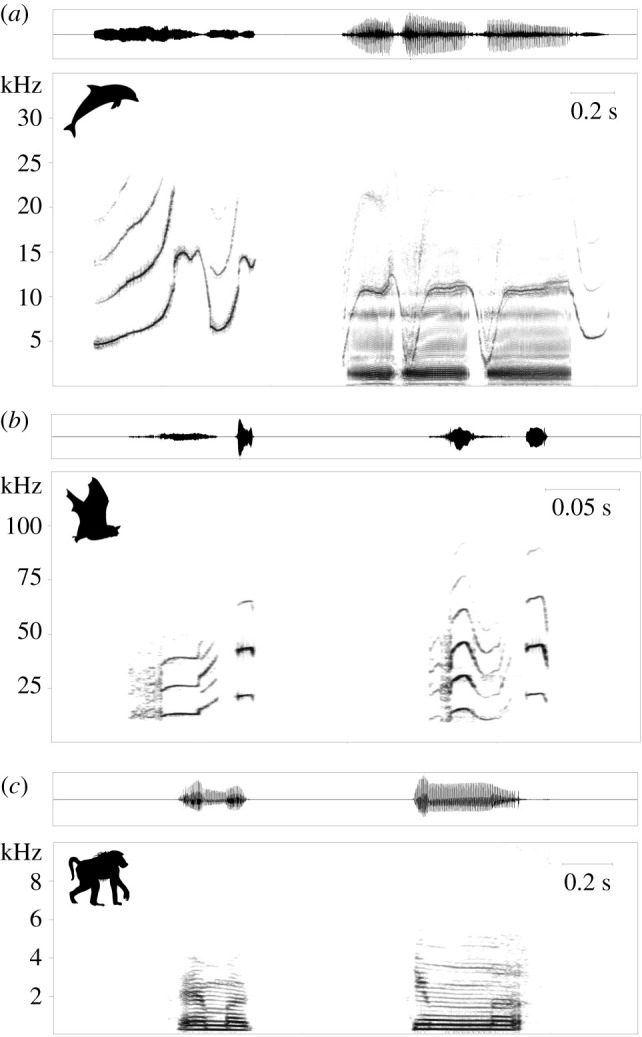
Waveforms and spectrograms of vocalizations produced by species with different capacities for vocal production learning. Call types from three taxa were selected to illustrate the degree of structural variability of acoustic signals covered in this review. (*a*) Strikingly different signature whistles from two common bottlenose dolphins, *Tursiops truncatus*. (*b*) Moderately different isolation calls from two greater sac-winged bats, *Saccopteryx bilineata*. (*c*) Subtly different grunts from two male Guinea baboons, *Papio papio* (courtesy of J. Fischer). Note that different taxa have different sound production mechanisms. Spectrograms were generated with a 1024-point FFT and a Hamming Window with 75% (*b*) or 87.5% (*a*,*c*) overlap, a sampling rate of 80 (*a*), 300 (*b*) or 41 (*c*) kHz and a resolution of 16 bits.

Good evidence for vocal production learning in toothed whales comes from bottlenose dolphins (*Tursiops truncatus*). The most convincing reports demonstrating this ability are training studies in which animals were conditioned to copy tonal, computer-generated model sounds [18,19]. While pre-test repertoires were not presented, some of the models were unlike dolphin whistles described before, including abrupt frequency changes between unmodulated tones and instantaneous changes in the direction of frequency modulations. The animals were able to copy such whistles with good accuracy. More recent studies focused on how learning influences whistle development in this species. Bottlenose dolphins develop individually distinctive signature whistles [20,21] (figure 2*a*) which are novel and distinctive frequency modulation patterns broadcasting the identity of the caller [22]. Fripp *et al*. [23] found that signature whistles of bottlenose dolphins in the wild were more similar to those of members of their population than to whistles of a captive colony. This suggests the use of vocal learning in signature whistle development, possibly by using a model and then changing it to achieve distinctiveness. However, differences between captive and wild dolphin whistles could be caused by other factors. Miksis *et al*. [24] showed that signature whistles of captive dolphins seem to contain parts resembling the constant-frequency bridge whistles used by animal-care staff during training. This shows that there can be a consistent difference between captive and wild dolphin whistles, most likely due to vocal production learning influencing whistles depending on the acoustic environment. Learning also likely plays a role in whistle matching in which different animals produce the same whistle type in quick succession [25]. Signature whistles are often used in these interactions [26]. Since every animal develops its own novel and distinctive signature whistle, vocal production learning appears to be the only way in which others could acquire these whistles to use in matching interactions.

Other delphinid species may have similar skills but only a few have been studied to date. One example is an orphaned, captive Risso's dolphin (*Grampus griseus*) that was found to produce whistles more similar to those of bottlenose dolphins it was housed with than to whistles of wild Risso's dolphins [27]. Vocal production learning in the largest delphinid, the killer whale (*Orcinus orca*), has been studied in more detail. Adamson *et al*. [28] performed a learning experiment similar in design to the training studies on bottlenose dolphins in which animals were trained to copy model sounds consisting of human words and sounds of other killer whales. While the killer whale's copies resembled several parameters of model sounds, copying accuracy was surprisingly low in others when compared to vocal learning studies on other species. An important component in vocal production learning studies is a quantitative comparison of model sounds with the existing repertoire of the animal before tests begin, especially when copies are of low fidelity. This is important to demonstrate production learning rather than usage learning. Such an analysis was part of this study, but the similarity was only assessed by a human judge and not by the quantitative methods used to identify copies in this study. Another study focused on changes in the repertoire of calls used by captive killer whales and found that the use of calls in juvenile whales changed over time and that they learned new call types [29]. Two juvenile males born in captivity started to associate with and produce calls of an unrelated, adult male over the course of the study.

In another captive study, killer whales housed with bottlenose dolphins also changed their click and whistle patterns so that they resembled those produced by bottlenose dolphins, a good indicator for usage learning [30]. In addition, one animal started to produce a repeated whistle that the bottlenose dolphins were trained to produce for public shows. While trainers had reported that the killer whale did not produce such sequences before, it is unclear whether the relatively simple modulation pattern of the whistle in the sequence was already present in the whale's repertoire before. Thus, this study provides good evidence for usage learning but is not conclusive on production learning.

In the wild, killer whales have been found to copy calls of other pods (reviewed in [3,5]) but it is not always clear whether these are cases of usage or production learning. A wild killer whale apparently copying a California sea lion (*Zalophus californianus*) has also been reported [31]. The assumed copies had harmonics above 4 kHz which are usually not found in sea lion barks underwater but a quantitative analysis of similarity was not provided and distant sea lions could have been responsible. Perhaps the most convincing evidence for killer whale production learning in the wild comes from observations that members from different pods have been observed to change the structure of a shared call type in parallel over a 12-year period while leaving another analysed call type unchanged [32]. This gradual change is similar to that found in songs of Northern Hemisphere humpback whales (*Megaptera novaeangliae*) [33] and provides evidence that learning is taking place.

Vocal learning in belugas (*Delphinapterus leucas*) had been described anecdotally in the past [3], but more detailed studies have been published since. Ridgway *et al*. [34] reported sounds that appeared to mimic human speech sounds in a trained beluga. These copies sounded distorted and model words could not be recognized, but the authors stated that these sounds resembled human speech as heard underwater when divers communicated with each other. Unfortunately, a quantitative comparison was not provided. Murayama *et al*. [35] trained a beluga to copy model sounds presented to it. These sounds included human speech sounds and computer-generated tonal sounds. Even though all models were presented in air, the authors reported good copying skills, but only compared copies to all possible model sounds without a comparison with the pre-training repertoire of the animal. Panova & Agafonov [36] reported a beluga producing copies of bottlenose dolphin signature whistles after being kept with them in the same pool. The similarity between models and copies was high, but no recordings of the beluga from before the introduction or from control groups were available. In such cross-species copying tests, comparisons with baseline repertoires may seem less important. However, many mammals produce sounds similar to those of other species, including humans, in their repertoires, so that usage learning may influence these results to some extent.

Studying vocal learning in baleen whales is much more challenging. Nevertheless, male humpback whales appear to provide good evidence for vocal production learning in how they modify their song types over time [3]. In the Northern Hemisphere, song type change is slow but all males in a population produce the same song type at any one time [33], which seems difficult to achieve without learning. More recent studies in the Southern Hemisphere showed that a communal change in song type can be more rapid, with the entire song changing completely for all singers within little over a year [37,38]. Interestingly, different breeding aggregations represent distinct populations in the Southern Hemisphere but males have been observed to switch between them [39], a pattern prevented by land barriers in the Northern Hemisphere. Each of these populations has its own song type, but song types that are found in one population tend to be picked up by others to the east in later years [38]. Some authors suggest that humpback whales may have a finite number of song elements that they recombine through usage learning [40–42] but considering the gradual changes in song types especially in the Northern Hemisphere, vocal production learning seems to be a more likely explanation for the changes observed.

While early work on bowhead whales (*Balaena mysticetus*) around North America found a similar pattern of communal song type change from year to year as described for humpback whales, with just one song type in each season [43], more recent work has shown that bowheads can sing more than one song type per year. In Fram Strait, bowhead whales sang multiple song types in a season and appeared to share song types [44]. The occurrence of specific song types on an acoustic recorder in the area was almost sequential through the year and no song type was recorded in more than one season over an observation period of four years. Bowhead whale singing patterns, while different from humpback whales, would be difficult to explain without evoking vocal production learning.

## 3. Pinnipeds

Pinnipeds are well known for their trainability in captivity and a number of studies have shown that they are capable of usage learning as demonstrated by conditioned production of calls in their repertoire [45,46]. Training studies on harbour seals (*Phoca vitulina*) [45] and walrus (*Odobenus rosmarus*) [47] have also shown that they can invent calls as judged by a trainer and increase the variability of their calls when rewarded accordingly. Evidence for vocal production learning in pinnipeds comes from studies using human speech as templates. An early observational study on two harbour seals (*Phoca vitulina*) showed that they could acquire human words when raised by human caretakers [48]. A study on three grey seals (*Halichoerus grypus*) investigated this ability in phocid seals experimentally, training one animal to copy melodies and two to copy human vowels [49]. The animals shifted the formants in their calls to achieve vowel matches, which constitutes filter learning. All seals were recorded from birth and could also be trained to produce seal calls at frequencies outside of their baseline repertoire or the repertoire described for the species. A subsequent study on wild grey seal pups demonstrated that vocal production learning influenced the development of their pup calls. Independent groups of animals copied unknown frequency modulation sequences and individual calls played back to them depending on the kind of playback they were exposed to [50].

Observational evidence in the wild is harder to come by. While geographic variation is common in phocid seal calls [7], a detailed study of what appeared to be Northern elephant seal (*Mirounga angustirostris*) dialects turned out to be the result of a population bottleneck and founder effect [51]. The same breeding rookeries recorded nearly 50 years later revealed that the differences found between them in the earlier studies had disappeared, replaced by a much greater call variability between males than found before [52]. Nevertheless, one study on Southern elephant seals (*Mirounga leonina*) provided evidence that males learn at least temporal parameters of their dominance calls from successful conspecifics in wild breeding aggregations [53].

## 4. Elephants

Elephants are a relatively new addition to the list of mammalian vocal learners. A first report documented an African elephant (*Loxodonta africana*) kept with Asian elephants (*Elephas maximus*) producing chirping sounds similar to those of Asian elephants, and an adolescent African elephant in an orphanage copying the duration and frequency bandwidth of truck sounds [54]. In both cases, copies were more similar to the model than to other conspecifc sounds and involved modifying frequency as well as temporal parameters. Another study described the vocalizations of a single Asian elephant copying human speech sounds in a zoo [55]. In this latter example, the animal used its trunk to change the shape of its mouth cavity to copy human vowels. Wild elephants are not known to modify vocalizations this way, making this a very unusual example of vocal copying mediated not by control over the vocal production apparatus but over trunk musculature.

## 5. Bats

Echolocating greater horseshoe bats (*Rhinolophus ferrumequinum*) emit a very narrowly defined resting frequency (RF) in the prominent constant-frequency component of their echolocation calls, which is different in experimentally deafened individuals [56]. An observational long-term study showed that the RF decreases over an individual's lifetime in the wild [57]. Interestingly, in recordings of echolocation calls of mother–pup pairs, a pup's RF was similar to the current RF of its mother [57], indicating call convergence. The RF of mothers and pups were correlated, and pups of young mothers had a higher RF than pups of the same mothers later in life. Correspondingly, RF in the Taiwanese leaf-nosed bat, *Hipposideros terasensis*, appears to be influenced by conspecifics. Bats that were experimentally transferred to a new colony adjusted their RF after 8–16 days to the resident bats' RF [58]. However, it is unclear if transfer-induced stress may have affected the bats’ RF.

Greater spear-nosed bat (*Phyllostomus hastatus*) females produce noisy screech calls which encode a group-specific signature to facilitate group cohesion during foraging [59,60]. The group signature results from a call convergence of all group members [61]. In a transfer experiment, captive subadult females were assigned to two new social groups, replicating the dispersal pattern of subadult females in the wild. The screech calls of all group members changed mainly in peak frequency and spectral shape, converging to maintain two different group-specific vocal signatures. As the potential effects of maturation, physical environment or genetic relatedness on call convergence were controlled for, auditory input from conspecifics appears to be the crucial factor for the acquisition of the observed group-specific signatures. Such convergence within but not between highly controlled groups can indicate vocal production learning if usage learning can be excluded.

In an experiment on pale spear-nosed bats (*Phyllostomus discolor*), adult bats were trained to match an auditory target (a frequency-shifted social call from their repertoire) which required them to lower the fundamental frequency of their social calls [62]. Once lowering the fundamental frequency was no longer required to receive a reward, most bats resumed calling with higher fundamental frequencies. One individual, however, raised the fundamental frequency of its calls again only after the frequency of the auditory target was raised as well, thus demonstrating it paid attention to the auditory experience provided by the target.

Captive, adult Egyptian fruit bats, *Rousettus aegyptiacus*, exposed to continuous broadband noise for two weeks reacted by shifting their call frequency upwards [63]. This shift was persistent for several weeks after noise cessation, suggesting that adult bats showed vocal plasticity. This plasticity could be caused by vocal usage learning or vocal production learning. In a different study, pups raised in relative acoustic isolation (i.e. only with their mothers) had a delayed vocal repertoire maturation, producing calls with a higher fundamental frequency and greater variability than control pups that were raised with auditory feedback from more co-housed conspecifics [64]. In the same study, three additional pups raised in isolation but with exposure to playbacks of low-frequency adult calls also produced the lower frequency calls. However, differences in isolation studies are often difficult to interpret and hearing adult calls could simply reduce stress levels and facilitate normal vocal development. In a follow-up study, pups housed only with their mothers were raised with three different playbacks of conspecific calls that differed in their fundamental frequency [65]. This experiment demonstrated call convergence in which pups produced calls with different fundamental frequencies depending on their auditory input. This convergence towards different sound types is consistent with vocal production learning but difficult to interpret because all analysed sound types were pooled and not considered separately. It is possible that bats instead chose different call types from their existing repertoires to achieve this outcome.

Greater sac-winged bat (*Saccopteryx bilineata*) pups produce multisyllabic isolation calls which encode individual and group-specific differences (figure 2*b*). In an observational study on captive groups, the isolation calls of free-living pups from seven different social groups converged in spectral composition towards the isolation calls of their respective group members [66]. As potentially confounding effects on call convergence were ruled out (i.e. maturation, physical environment and genetic relatedness), auditory input from conspecifics appears to be the crucial factor for the group-specific signature. Pups of both sexes not only produced isolation calls but also very long ‘babbling bouts’, i.e. sequences containing precursors of most adult syllable types [67]. One conspicuous adult vocalization type, the multisyllabic territorial song of males, first appeared in rudimentary form in pups' babbling bouts and subsequently transformed into fully developed song, showing the same syntactical and spectral composition as the adult song [68]. The territorial song consists of up to six different syllable types, the most prominent being the buzz syllable. Regarding their spectral characteristics, buzz syllables of free-living pups from seven different social groups became more similar to, and finally strongly resembled, the buzz syllables from adult males belonging to the pups’ respective social group but not to other social groups in the vicinity. When pups produced buzz syllables for the first time, they had already been exposed to singing males for two to three weeks. This auditory input guided the pups' attempts to copy the male song, a task they mastered after another seven to nine weeks. The observed similarity of buzz syllables from pups and adult males was irrespective of whether pups were related to singing males or not, thus demonstrating the importance of auditory input and hence vocal production learning for song acquisition. Intriguingly, pups of both sexes learned to sing even though only males sing as adults. Overall, the process of copying tutor song and the pronounced vocal practice phase in *S. bilineata* shows interesting parallels to song learning in oscine songbirds.

## 6. Primates

Primates have long been a major focus for studies on vocal learning. Clearly humans have advanced vocal learning skills and we would therefore expect to find this in other primates as well. However, evidence for the occurrence of vocal production learning in nonhuman primates has not been forthcoming. From a considerable body of work, it is clear that vocal learning abilities in nonhuman primates are much more limited than in humans. In all cases of nonhuman primate vocal modifications, it appears that animals produced altered versions of calls that were already in their repertoire.

The production of such novel signals has been reported in captive primates using filter structures such as lips, cheeks and the tongue. Koko, a western lowland gorilla (*Gorilla gorilla*), raised and cared for by humans without other gorillas produced a large repertoire of such sounds such as blows, huffs and coughs [69]. Similarly, ten orangutans (*Pongo* spp.) in human care have learned a whistle and two of them matched temporal parameters of whistles produced by humans [70]. One orangutan also managed to match aspects of frequency modulation in a so-called wookie call. These calls have been described in captive animals but they strongly overlap with calls in the natural repertoire of orangutans and their production involves the larynx [71]. In a training experiment, the same orangutan produced high- and low-frequency versions of the wookie call in response to high- and low-frequency versions produced by humans. While the frequencies used by humans and organutans did not match, this behaviour suggests the animal was trying to copy the human model [71]. Wild orangutans also appear to have greater geographic variation in call structure than other species [72] but learning has not yet been demonstrated as a cause of this variation.

The main body of recent evidence for vocal flexibility in nonhuman primates comes from studies on vocal convergence within call types (figure 1). Several studies reported greater acoustic similarities between closely associated animals that were not genetically related than between non-associates, including grey mouse lemurs (*Microcebus murinus*) [73], Campbell's monkeys (*Cercopithecus campbelli*) [74], Guinea baboons (*Papio papio*) [75] (figure 2*c*) and chimpanzees (*Pan troglodytes*) [76]. To further investigate the process of convergence, studies have documented call characteristics before and after housing animals with previously unknown conspecifics. In pygmy marmosets (*Cebuella pygmaea*), three out of four individuals showed call convergence after being paired with a new animal [77]. Similarly, trill and phee calls of eight common marmosets (*Callithrix jacchus*) became more similar between two groups once they had been placed into acoustic contact [78]. Thirteen chimpanzees showed convergence over several years in their food grunts after being housed together [79]. All of these subtle changes were consistent and long-lasting. Short-term convergence of calls in social interactions has also been found in interactions of chimpanzees [80] and Diana monkeys (*Cercopithecus diana*) [81]. However, as mentioned before, subtle changes within call types can also be caused by other effects, especially in the wild where group composition and environmental factors are not controlled for. For food call convergence in chimpanzees [79], it has been convincingly argued that the reported new variants of food calls were already in the animals repertoires before a change in use was observed, which would make this an example of usage learning [82]. Furthermore, changes in acoustic parameters can be caused by changes in arousal or motivational state over time in which case learning does not need to be involved [82]. While there were good arguments to exclude arousal changes as an explanation in the chimpanzee example [83], it is a valid alternative explanation in many cases of subtle vocal changes. Nevertheless, these cases of convergence are intriguing and further work on the mechanisms behind them are needed to assess when vocal learning might be involved [84].

Many recent studies also provide examples of primate vocal plasticity in other domains. Usage learning has been reconfirmed with more advanced methodology in recent studies in which common marmosets were trained to produce calls from their repertoire in response to conditioned signals [85] and chimpanzees found to incorporate raspberry sounds into their call sequences [86]. Furthermore, captive chimpanzees learned to use sounds to get attention from humans, and which sounds they used could be conditioned with selective rewards [87]. Finally, a lack of consistency in vocal responses by parents can delay the vocal development in common marmosets [88]. All of these examples show flexibility in vocalizations. Yet, the overall structure of nonhuman primate vocalizations has been shown to be comparatively stable within species [89].

## 7. Other mammalian orders

As in primates, vocal convergence in other mammalian taxa leads to more subtle acoustic changes than have been reported for cetaceans, pinnipeds, elephants and bats. Naked mole-rats (*Heterocephalus glaber*) modify the frequency modulation of their most common call type, the soft chirp, based on the auditory input from conspecifics they grow up with [90]. Naked mole-rats live in multigenerational, eusocial colonies. Soft chirps function as contact calls and encode a group-specific signature that mediates antiphonal calling between group members. Experimentally transferred pups adopt the signature of their foster colony, indicating call convergence which is not driven by genetic relatedness. Moreover, the colony signature in soft chirps deteriorates when the matriarch of a colony is replaced, further highlighting the importance of conspecific influences on call convergence.

Good evidence for convergence was also reported for ungulates. The contact calls of captive pygmy goat (*Capra hircus*) kids converged towards the calls of fellow kids in four different social group over the course of 35 days after birth [91]. Call convergence led to changes in fundamental frequency and formant structure. When assessing the group-specific signature, genetic and environmental effects were controlled for and could not explain this pattern.

Male common house mice (*Mus musculus*) from one genetic strain, B6, decreased the peak frequency of their songs' dominant syllable towards that of males from another strain, BxD, when housed under competitive social conditions (i.e. one male from each strain together with one female from the one or the other strain) [92]. The behavioural function of the observed shift is unclear and stress could have influenced call changes. While it is possible that mouse song is influenced by the acoustic environment, other studies clearly demonstrated that mice do not need auditory input for song development. Both genetically deaf mice [93] and experimentally deafened mice [94] developed normal song and cross-fostering did not influence song characteristics [95]. Correspondingly, a mouse strain lacking its cerebral cortex also developed normal song [96], indicating that song production in mice is controlled by subcortical structures such as the striatum and the midbrain.

## 8. Conclusion

Since the last review [3], a considerable number of studies have reported new results on vocal learning in mammals. With more detailed evidence available, it becomes apparent that vocal production learning is not an all or nothing skill but that it can influence vocal behaviour to different degrees. Looking forward, the key issue to address is the variability in learning skills between species. For this, we need to find a standardized approach to mapping out an animal's acoustic space, i.e. the kinds of sounds its production apparatus could theoretically produce and compare it to limitations when it comes to copying sounds. Training methods based on vocalizations already present in an animal's repertoire that then get modified once the subject learns to associate a specific modification with a reward (e.g. [49]), may be the way ahead. An alternative or complementary approach may be the more exact analysis of copied sound patterns in the wild, especially in species that are not easily trained such as large whales. It is apparent that the vocal production learning abilities of cetaceans, seals, elephants and some bats are more pronounced than the examples of subtle convergence within call types found in other orders (figure 1). Convergence usually only requires comparatively minor adjustments, so that usage learning appears sufficient to achieve them. Alternatively, such convergence may be a result of a shared physiological state not requiring learning. Nevertheless, minor changes may still be mediated by direct connections between the neocortex and the vocal production apparatus and deserve further study. Whether convergence and other subtle adjustments use the same neural mechanisms as vocal production learning is one of the key questions in this field (Vernes *et al*. [97]). Only by focusing on the degree of sharing in mechanisms will we be able to classify learning patterns in a biologically sensible way. If the same mechanism is used, differences may only occur in degree but not in kind of learning.

We did not revisit the contexts in which learned sounds are used or in which vocal learning may have evolved. These have not changed fundamentally [98] since the review by Janik & Slater [3] (but see Caruso *et al*. [99] for a broader look at contexts). Vocal convergence in the development of social relationships and potential adjustments animals make to cope with added noise in the environment have been highlighted as possible additional contexts for vocal learning [84]. Convergence in the context of social bonds has been included by Janik & Slater [3] in recognition contexts but may deserve separate consideration. Furthermore, adjustments to noise may have paved the way for greater vocal control and could have been a stepping stone in the evolution of vocal learning [84]. Alternatively, such reactions could be genetically encoded with little influence from learning. Further study is needed to make these distinctions.

One of the main outcomes of bringing together all the evidence for or against a particular trait is a recognition of different methods and approaches used in its study. A common theme in studies on vocal learning is the often superficial treatment of comparisons to the existing repertoire. Sometimes data from before tests began are not available, but a substitute can be the comparison with the species repertoire in general. Unfortunately, such comparisons are often not as detailed as those used when trying to demonstrate similarities between a model and a match. Comparisons to before tests started or to the species repertoire are crucial when trying to decide whether vocal production learning leads to the rise of a new vocalization or whether the animal uses already existing calls or songs to achieve a match through usage learning. The general conclusion from our revisit of this subject though is that the increased number of studies on vocal production learning in mammals helps to confirm the degree to which vocal learning is present in each particular order (figure 1). Repeated studies showing advanced or limited learning skills help to paint the picture of how vocal learning has evolved and what its role is in the complexity of each species' communication system.

## Data accessibility

This article has no additional data.

## References

[1] Janik VM, Slater PJB. 2000 The different roles of social learning in vocal communication. Anim. Behav. **60**, 1-11. (10.1006/anbe.2000.1410) 10924198

[2] Catchpole CK, Slater PJB. 2008 Bird song: biological themes and variations, 2nd edn. Cambridge, UK: Cambridge University Press.

[3] Janik VM, Slater PJB. 1997 Vocal learning in mammals. Adv. Study Behav. **26**, 59-99. (10.1016/S0065-3454(08)60377-0)

[4] Janik VM, Slater PJB. 2003 Traditions in mammalian and avian vocal communication. In The biology of traditions: models and evidence (eds DM Fragaszy, S Perry), pp. 213-235. Cambridge, UK: Cambridge University Press.

[5] Janik VM. 2014 Cetacean vocal learning and communication. Curr. Opin. Neurobiol. **28**, 60-65. (10.1016/j.conb.2014.06.010) 25057816

[6] Knörnschild M. 2014 Vocal production learning in bats. Curr. Opin. Neurobiol. **28**, 80-85. (10.1016/j.conb.2014.06.014) 25050812

[7] Reichmuth C, Casey C. 2014 Vocal learning in seals, sea lions, and walruses. Curr. Opin. Neurobiol. **28**, 66-71. (10.1016/j.conb.2014.06.011) 25042930

[8] Stoeger AS, Manger P. 2014 Vocal learning in elephants: neural bases and adaptive context. Curr. Opin. Neurobiol. **28**, 101-107. (10.1016/j.conb.2014.07.001) 25062469 PMC4181794

[9] Elemans CPH et al. 2015 Universal mechanisms of sound production and control in birds and mammals. Nat. Commun. **6**, 8978. (10.1038/ncomms9978) 26612008 PMC4674827

[10] Reidenberg JS, Laitman JT. 2018 Anatomy of underwater sound production with a focus on ultrasonic vocalization in toothed whales including dolphins and porpoises. In Handbook of ultrasonic vocalization: a window into the emotional brain, vol. 25 (eds SM Brudzynski), pp. 509-519. Amsterdam, The Netherlands: Elsevier.

[11] Stoeger AS. 2021 Elephant sonic and infrasonic sound production, perception, and processing. In Neuroendocrine regulation of animal vocalization: mechanisms and anthropogenic factors in animal communication (eds CS Rosenfeld, F Hoffmann), pp. 189-199. London, UK: Academic Press.

[12] MacNeilage PF. 2008 The origin of speech. Oxford, UK: Oxford University Press.

[13] Petkov CI, Jarvis ED. 2012 Birds, primates, and spoken language origins: behavioral phenotypes and neurobiological substrates. Front. Evol. Neurosci. **4**, 12. (10.3389/fnevo.2012.00012) 22912615 PMC3419981

[14] Scharff C, Knörnschild M, Jarvis E. 2019 Vocal learning and spoken language: insights from animal models with an emphasis on genetic contributions. In Human language: from genes and brains to behavior (eds P. Hagoort), pp. 657-686. Cambridge, MA: MIT Press.

[15] Fitch WT, Neubauer J, Herzel H. 2002 Call out of chaos: the adaptive significance of nonlinear phenomena in mammalian vocal production. Anim. Behav. **63**, 407-418. (10.1006/anbe.2001.1912)

[16] Wilden I, Herzel H, Peter G, Tembrock G. 1998 Subharmonics, biphonation, and deterministic chaos in mammal vocalization. Bioacoustics **9**, 171-196. (10.1080/09524622.1998.9753394)

[17] Zollinger SA, Podos J, Nemeth E, Goller F, Brumm H. 2012 On the relationship between, and measurement of, amplitude and frequency in bird song. Anim. Behav. **84**, e1-e9. (10.1016/j.anbehav.2012.04.026)

[18] Richards DG, Wolz JP, Herman LM. 1984 Vocal mimicry of computer-generated sounds and vocal labeling of objects by a bottlenosed dolphin, *Tursiops truncatus*. J. Comp. Psychol. **98**, 10-28. (10.1037/0735-7036.98.1.10) 6705501

[19] Sigurdson J. 1993 Frequency-modulated whistles as a medium for communication with the bottlenose dolphin (*Tursiops truncatus*). In Language and communication: comparative perspectives (eds HL Roitblat, LM Herman, PE Nachtigall), pp. 153-173. Hillsdale, NJ: Lawrence Erlbaum Associates.

[20] Caldwell MC, Caldwell DK, Tyack PL. 1990 Review of the signature-whistle-hypothesis for the Atlantic bottlenose dolphin. In The bottlenose dolphin (eds S Leatherwood, RR Reeves), pp. 199-234. San Diego, CA: Academic Press.

[21] Janik VM, Sayigh LS. 2013 Communication in bottlenose dolphins: 50 years of signature whistle research. J. Comp. Physiol. A **199**, 479-489. (10.1007/s00359-013-0817-7) 23649908

[22] Janik VM, Sayigh LS, Wells RS. 2006 Signature whistle contour shape conveys identity information to bottlenose dolphins. Proc. Natl Acad. Sci. USA **103**, 8293-8297. (10.1073/pnas.0509918103) 16698937 PMC1472465

[23] Fripp D, Owen C, Quintana-Rizzo E, Shapiro A, Buckstaff K, Jankowski K, Wells R, Tyack P. 2005 Bottlenose dolphin (*Tursiops truncatus*) calves appear to model their signature whistles on the signature whistles of community members. Anim. Cogn. **8**, 17-26. (10.1007/s10071-004-0225-z) 15221637

[24] Miksis JL, Tyack PL, Buck JR. 2002 Captive dolphins, *Tursiops truncatus*, develop signature whistles that match acoustic features of human-made model sounds. J. Acoust. Soc. Am. **112**, 728-739. (10.1121/1.1496079) 12186052

[25] Janik VM. 2000 Whistle matching in wild bottlenose dolphins (*Tursiops truncatus*). Science **289**, 1355-1357. (10.1126/science.289.5483.1355) 10958783

[26] King SL, Sayigh LS, Wells RS, Fellner W, Janik VM. 2013 Vocal copying of individually distinctive signature whistles in bottlenose dolphins. Proc. R. Soc. B **280**, 20130053. (10.1098/rspb.2013.0053) PMC3619487 23427174

[27] Favaro L, Neves S, Furlati S, Pessani D, Martin V, Janik VM. 2016 Evidence suggests vocal production learning in a cross-fostered Risso's dolphin (*Grampus griseus*). Anim. Cogn. **19**, 847-853. (10.1007/s10071-016-0961-x) 26874843

[28] Abramson JZ, Hernandez-Lloreda V, Garcia L, Colmenares F, Aboitiz F, Call J. 2018 Imitation of novel conspecific and human speech sounds in the killer whale (*Orcinus orca*). Proc. R. Soc. B **285**, 20172171. (10.1098/rspb.2017.2171) PMC5805929 29386364

[29] Crance JL, Bowles AE, Garver A. 2014 Evidence for vocal learning in juvenile male killer whales, *Orcinus orca*, from an adventitious cross-socializing experiment. J. Exp. Biol. **217**, 1229-1237. (10.1242/jeb.094300) 24744421

[30] Musser WB, Bowles AE, Grebner DM, Crance JL. 2014 Differences in acoustic features of vocalizations produced by killer whales cross-socialized with bottlenose dolphins. J. Acoust. Soc. Am. **136**, 1990-2002. (10.1121/1.4893906) 25324098

[31] Foote AD, Griffin RM, Howitt D, Larsson L, Miller PJO, Hoelzel AR. 2006 Killer whales are capable of vocal learning. Biol. Lett. **2**, 509-512. (10.1098/rsbl.2006.0525) 17148275 PMC1834009

[32] Deecke VB, Ford JKB, Spong P. 2000 Dialect change in resident killer whales: implications for vocal learning and cultural transmission. Anim. Behav. **60**, 629-638. (10.1006/anbe.2000.1454) 11082233

[33] Payne K, Payne R. 1985 Large scale changes over 19 years in songs of humpback whales in Bermuda. Z. Tierpsychol. **68**, 89-114. (10.1111/j.1439-0310.1985.tb00118.x)

[34] Ridgway S, Carder D, Jeffries M, Todd M. 2012 Spontaneous human speech mimicry by a cetacean. Curr. Biol. **22**, R860-R861. (10.1016/j.cub.2012.08.044) 23098588

[35] Murayama T, Iijima S, Katsumata H, Arai K. 2014 Vocal imitation of human speech, synthetic sounds and beluga sounds, by a beluga (*Delphinapterus leucas*). Int. J. Comp. Psychol. **27**, 369-384. (10.46867/ijcp.2014.27.03.10)

[36] Panova EM, Agafonov AV. 2017 A beluga whale socialized with bottlenose dolphins imitates their whistles. Anim. Cogn. **20**, 1153-1160. (10.1007/s10071-017-1132-4) 28956181

[37] Noad MJ, Cato DH, Bryden MM, Jenner MN, Jenner KCS. 2001 Cultural revolution in whale song. Nature **408**, 537. (10.1038/35046199) 11117730

[38] Garland EC, Goldizen AW, Rekdahl ML, Constantine R, Garrigue C, Hauser ND, Poole MM, Robbins J, Noad MJ. 2011 Dynamic horizontal cultural transmission of humpback whale song at the ocean basin scale. Curr. Biol. **21**, 687-691. (10.1016/j.cub.2011.03.019) 21497089

[39] Garland EC et al. 2015 Population structure of humpback whales in the western and central South Pacific Ocean as determined by vocal exchange among populations. Conserv. Biol. **29**, 1198-1207. (10.1111/cobi.12492) 25851618

[40] Cerchio S, Jakobsen JK, Norris TF. 2001 Temporal and geographical variation in songs of humpback whales, *Megaptera novaeangliae*: synchronous change in Hawaiian and Mexican breeding assemblages. Anim. Behav. **62**, 313-329. (10.1006/anbe.2001.1747)

[41] Garland EC, McGregor PK. 2020 Cultural transmission, evolution, and revolution in vocal displays: insights from bird and whale song. Front. Psychol. **11**, 544929. (10.3389/fpsyg.2020.544929) 33132953 PMC7550662

[42] Mercado EI. 2021 Song morphing by humpback whales: cultural or epiphenomenal? Front. Psychol. **11**, 574403. (10.3389/fpsyg.2020.574403) 33519588 PMC7844363

[43] Würsig B, Clark C. 1993 Behavior. In The bowhead whale (eds JJ Burns, JJ Montague, CJ Cowles), pp. 157-199. Lawrence, KS: Society of Marine Mammalogy.

[44] Stafford KM, Lydersen C, Wiig O, Kovacs KM. 2018 Extreme diversity in the songs of Spitsbergen's bowhead whales. Biol. Lett. **14**, 20180056. (10.1098/rsbl.2018.0056) 29618521 PMC5938564

[45] Schusterman RJ. 2008 Vocal learning in mammals with special emphasis on pinnipeds. In Evolution of communicative flexibility (eds DK Oller, U Griebel), pp. 41-70. Cambridge, MA: MIT Press.

[46] Stansbury AL, de Freitas M, Wu G-M, Janik VM. 2015 Can a gray seal (*Halichoerus grypus*) generalize call classes? J. Comp. Psychol. **129**, 412-420. (10.1037/a0039756) 26460856

[47] Schusterman RJ, Reichmuth C. 2008 Novel sound production through contingency learning in the Pacific walrus (*Odobenus rosmarus divergens*). Anim. Cogn. **11**, 319-327. (10.1007/s10071-007-0120-5) 18038276

[48] Ralls K, Fiorelli P, Gish S. 1985 Vocalizations and vocal mimicry in captive harbor seals, *Phoca vitulina*. Can. J. Zool. **63**, 1050-1056. (10.1139/z85-157)

[49] Stansbury A, Janik VM. 2019 Formant modification through vocal production learning in gray seals. Curr. Biol. **29**, 2244-2249. (10.1016/j.cub.2019.05.071) 31231051

[50] Stansbury AL, Janik VM. 2021 The role of vocal learning in call acquisition of wild grey seal pups. Phil. Trans. R. Soc. B **376**, 20200251. (10.1098/rstb.2020.0251) 34482728 PMC8419579

[51] Le Boeuf BJ, Petrinovich LF. 1974 Dialects of northern elephant seals, *Mirounga angustirostris*: origin and reliability. Anim. Behav. **22**, 565-663. (10.1016/S0003-3472(74)80013-8)

[52] Casey C, Reichmuth C, Costa DP, Le Boeuf B. 2018 The rise and fall of dialects in northern elephant seals. Proc. R. Soc. B **285**, 20182176. (10.1098/rspb.2018.2176) PMC6283944 30487313

[53] Sanvito S, Galimberti F, Miller EH. 2007 Observational evidence of vocal learning in southern elephant seals: a longitudinal study. Ethology **113**, 137-146. (10.1111/j.1439-0310.2006.01306.x)

[54] Poole JH, Tyack PL, Stoeger-Horwath AS, Watwood S. 2005 Elephants prove capable of vocal learning. Nature **434**, 455-456. (10.1038/434455a) 15791244

[55] Stoeger AS, Mietchen D, Oh S, de Silva S, Herbst CT, Kwon S, Fitch WT. 2012 An Asian elephant imitates human speech. Curr. Biol. **22**, 2144-2148. (10.1016/j.cub.2012.09.022) 23122846 PMC3548412

[56] Rübsamen R, Schäfer M. 1990 Audiovocal interactions during development? Vocalisation in deafened young horseshoe bats vs. audition in vocalisation impaired bats. J. Comp. Physiol. A **167**, 771-784. 2086791 10.1007/BF00189767

[57] Jones G, Ransome RD. 1993 Echolocation calls of bats are influenced by maternal effects and change over a lifetime. Proc. R. Soc. B **252**, 125-128. (10.1098/rspb.1993.0055) 8391702

[58] Hiryu S, Katsura K, Nagato T, Yamazaki H, Lin LK, Watanabe Y, Riquimaroux H. 2006 Intra-individual variation in the vocalized frequency of the Taiwanese leaf-nosed bat, *Hipposideros terasensis*, influenced by conspecific colony members. J. Comp. Physiol. A **192**, 807-815. (10.1007/s00359-006-0118-5) 16538514

[59] Wilkinson GS, Boughman JW. 1998 Social calls coordinate foraging in greater spear-nosed bats. Anim. Behav. **55**, 337-350. (10.1006/anbe.1997.0557) 9480702

[60] Boughman JW, Wilkinson GS. 1998 Greater spear-nosed bats discriminate group mates by vocalizations. Anim. Behav. **55**, 1717-1732. (10.1006/anbe.1997.0721) 9642014

[61] Boughman JW. 1998 Vocal learning by greater spear-nosed bats. Proc. R. Soc. B **265**, 227-233. (10.1098/rspb.1998.0286) PMC1688873 9493408

[62] Lattenkamp EZ, Vernes SC, Wiegrebe L. 2020 Vocal production learning in the pale spear-nosed bat, *Phyllostomus discolor*. Biol. Lett. **16**, 20190928. (10.1098/rsbl.2019.0928) 32289244 PMC7211467

[63] Genzel D, Desai J, Paras E, Yartsev MM. 2019 Long-term and persistent vocal plasticity in adult bats. Nat. Commun. **10**, 1-12. (10.1038/s41467-019-11350-2) 31358755 PMC6662767

[64] Prat Y, Taub M, Yovel Y. 2015 Vocal learning in a social mammal: demonstrated by isolation and playback experiments in bats. Sci. Adv. **1**, e1500019. (10.1126/sciadv.1500019) 26601149 PMC4643821

[65] Prat Y, Azoulay L, Dor R, Yovel Y. 2017 Crowd vocal learning induces vocal dialects in bats: playback of conspecifics shapes fundamental frequency usage by pups. PLoS Biol. **15**, e2002556. (10.1371/journal.pbio.2002556) 29088225 PMC5663327

[66] Knörnschild M, Nagy M, Metz M, Mayer F, von Helversen O. 2012 Learned vocal group signatures in the polygynous bat *Saccopteryx bilineata*. Anim. Behav. **84**, 761-769. (10.1016/j.anbehav.2012.06.029)

[67] Knörnschild M, Behr O, Von Helversen O. 2006 Babbling behavior in the sac-winged bat (*Saccopteryx bilineata*). Naturwissenschaften **93**, 451-454. (10.1007/s00114-006-0127-9) 16736178

[68] Knörnschild M, Nagy M, Metz M, Mayer F, von Helversen O. 2010 Complex vocal imitation during ontogeny in a bat. Biol. Lett. **6**, 156-159. (10.1098/rsbl.2009.0685) 19812069 PMC2865031

[69] Perlman M, Clark N. 2015 Learned vocal and breathing behavior in an enculturated gorilla. Anim. Cogn. **18**, 1165-1179. (10.1007/s10071-015-0889-6) 26139343

[70] Lameira AR, Hardus ME, Kowalsky B, de Vries H, Spruijt BM, Sterck EHM, Shumaker RW, Wich SA. 2013 Orangutan (*Pongo* spp.) whistling and implications for the emergence of an open-ended call repertoire: a replication and extension. J. Acoust. Soc. Am. **134**, 2326-2335. (10.1121/1.4817929) 23967963

[71] Lameira AR, Hardus ME, Mielke A, Wich SA, Shumaker RW. 2016 Vocal fold control beyond the species-specific repertoire in an orang-utan. Sci. Rep. **6**, 30315. (10.1038/srep30315) 27461756 PMC4962094

[72] Wich SA et al. 2012 Call cultures in orang-utans? PLoS ONE **7**, e36180. (10.1371/journal.pone.0036180) 22586464 PMC3346723

[73] Hafen T, Neveu H, Rumpler Y, Wilden I, Zimmermann E. 1998 Acoustically dimorphic advertisement calls separate morphologically and genetically homogenous populations of the grey mouse lemur (*Microcebus murinus*). Fol. Primatol. **69**, 342-356. (10.1159/000052723) 9595693

[74] Lemasson A, Ouattara K, Petit EJ, Zuberbuhler K. 2011 Social learning of vocal structure in a nonhuman primate? BMC Evol. Biol. **11**, 362. (10.1186/1471-2148-11-362) 22177339 PMC3260242

[75] Fischer J, Wegdell F, Trede F, Dal Pesco F, Hammerschmidt K. 2020 Vocal convergence in a multi-level primate society: insights into the evolution of vocal learning. Proc. R. Soc. B **287**, 20202531. (10.1098/rspb.2020.2531) PMC7779498 33323082

[76] Crockford C, Herbinger I, Vigilant L, Boesch C. 2004 Wild chimpanzees produce group-specific calls: a case for vocal learning? Ethology **110**, 221-243. (10.1111/j.1439-0310.2004.00968.x)

[77] Snowdon CT, Elowson AE. 1999 Pygmy marmosets modify call structure when paired. Ethology **105**, 893-908. (10.1046/j.1439-0310.1999.00483.x)

[78] Zürcher Y, Willems EP, Burkart JM. 2019 Are dialects socially learned in marmoset monkeys? Evidence from translocation experiments. PLoS ONE **14**, e0222486. (10.1371/journal.pone.0222486) 31644527 PMC6808547

[79] Watson SK, Townsend SW, Schel AM, Wilke C, Wallace EK, Cheng L, West V, Slocombe KE. 2015 Vocal learning in the functionally referential food grunts of chimpanzees. Curr. Biol. **25**, 495-499. (10.1016/j.cub.2014.12.032) 25660548

[80] Mitani JC, Gros-Louis J. 1998 Chorusing and call convergence in chimpanzees: tests of three hypotheses. Behaviour **135**, 1041-1064. (10.1163/156853998792913483)

[81] Candiotti A, Zuberbuhler K, Lemasson A. 2012 Convergence and divergence in Diana monkey vocalizations. Biol. Lett. **8**, 382-385. (10.1098/rsbl.2011.1182) 22237503 PMC3367761

[82] Fischer J, Wheeler BC, Higham JP. 2015 Is there any evidence for vocal learning in chimpanzee food calls? Curr. Biol. **25**, R1028-R1029. (10.1016/j.cub.2015.09.010) 26528740

[83] Watson SK, Townsend SW, Schel AM, Wilke C, Wallace EK, Cheng L, West V, Slocombe KE. 2015 Reply to Fischer *et al*. Curr. Biol. **25**, R1030-R1031. (10.1016/j.cub.2015.09.024) 26528741

[84] Tyack PL. 2008 Convergence of calls as animals form social bonds, active compensation for noisy communication channels, and the evolution of vocal learning in mammals. J. Comp. Psychol. **122**, 319-331. (10.1037/a0013087) 18729661

[85] Pomberger T, Risueno-Segovia C, Gultekin YB, Dohmen D, Hage SR. 2019 Cognitive control of complex motor behavior in marmoset monkeys. Nat. Commun. **10**, 3796. (10.1038/s41467-019-11714-8) 31439849 PMC6706403

[86] Marshall AJ, Wrangham RW, Arcadi AC. 1999 Does learning affect the structure of vocalizations in chimpanzees? Anim. Behav. **58**, 825-830. (10.1006/anbe.1999.1219) 10512656

[87] Taglialatela JP, Reamer L, Schapiro SJ, Hopjins WD. 2012 Social learning of a communicative signal in captive chimpanzees. Biol. Lett. **8**, 498-501. (10.1098/rsbl.2012.0113) 22438489 PMC3391466

[88] Takahashi DY, Liao DA, Ghazanfar AA. 2017 Vocal learning via social reinforcement by infant marmoset monkeys. Curr. Biol. **27**, 1844-1852. (10.1016/j.cub.2017.05.004) 28552359

[89] Fischer J, Hammerschmidt K. 2019 Towards a new taxonomy of primate vocal production learning. Phil. Trans. R. Soc. B **375**, 20190045. (10.1098/rstb.2019.0045) 31735147 PMC6895554

[90] Barker AJ, Veviurko G, Bennett NC, Hart DW, Mograby L, Lewin GR. 2021 Cultural transmission of vocal dialect in the naked mole-rat. Science **371**, 503-507. (10.1126/science.abc6588) 33510025

[91] Briefer EF, McElligott AG. 2012 Social effects on vocal ontogeny in an ungulate, the goat, *Capra hircus*. Anim. Behav. **83**, 991-1000. (10.1016/j.anbehav.2012.01.020)

[92] Arriaga G, Zhou EP, Jarvis ED. 2012 Of mice, birds, and men: the mouse ultrasonic song system has some features similar to humans and song-learning birds. PLoS ONE **7**, e46610. (10.1371/journal.pone.0046610) 23071596 PMC3468587

[93] Hammerschmidt K, Reisinger E, Westekemper K, Ehrenreich L, Srenzke N, Fischer J. 2012 Mice do not require auditory input for the normal development of their ultrasonic vocalizations. BMC Neurosci. **13**, 40. (10.1186/1471-2202-13-40) 22533376 PMC3350408

[94] Mahrt EJ, Perkel DJ, Tong L, Rubel EW, Portfors CV. 2013 Engineered deafness reveals that mouse courtship vocalizations do not require auditory experience. J. Neurosci. **33**, 5573-5583. (10.1523/JNEUROSCI.5054-12.2013) 23536072 PMC3691057

[95] Kikusui T, Nakanishi K, Nakagawa R, Nagasawa M, Mogi K, Okanoya K. 2011 Cross fostering experiments suggest that mice songs are innate. PLoS ONE **6**, e17721. (10.1371/journal.pone.0017721) 21408017 PMC3052373

[96] Hammerschmidt K, Whelan G, Eichele G, Fischer J. 2015 Mice lacking the cerebral cortex develop normal song: insights into the foundations of vocal learning. Sci. Rep. **5**, 8808. (10.1038/srep08808) 25744204 PMC4351519

[97] Vernes SC, Kriengwatana BP, Beeck VC, Fischer J, Tyack PL, ten Cate C, Janik VM. 2021 The multi-dimensional nature of vocal learning. Phil. Trans. R. Soc. B 376, 20200236. (10.1098/rstb.2020.0236) 34482723 PMC8419582

[98] Nowicki S, Searcy WA. 2014 The evolution of vocal learning. Curr. Opin. Neurobiol. **28**, 48-53. (10.1016/j.conb.2014.06.007) 25033109

[99] Carouso-Peck S, Goldstein M, Fitch T. 2021 The many functions of vocal learning. Phil. Trans. R. Soc. B **376**, 20200235. (10.1098/rstb.2020.0235) 34482721 PMC8419581

